# Supplementary material for: The secret life of baby turtles: A novel system to predict hatchling emergence, detect infertile nests, and remotely monitor sea turtle nest events
Source: PLoS One. 2022 Oct 26;17(10):e0275088. doi: 10.1371/journal.pone.0275088 (PMC9605334; doi:10.1371/journal.pone.0275088)
Supplement: Attachment — Submitted filename: reviewer response AUG5_2022.docx [file pone.0275088.s003.docx]

August 5, 2022

Dear PLoS ONE,

Thank you to the reviewer for your careful reading of the manuscript. We find your help extremely useful and are grateful for your time. We have made all requested changes, as listed below.

Thank you,

Erin Clabough

Line 56) “almost” to the day
*This change has been made.*

Lines 58 - 59) It is more appropriate to say “Our results suggest that motion plays a large role in…. instead of saying you hypothesise as it is from your findings that bring you to this.

*This change has been made.*

Same on Line 650-652. Suggested change “As motion ceases within the nest, it is possible that this final quieting down might be the cue to hatchlings that all their siblings have hatched and it is time to leave the nest”

*This change has been made.*

Lines 70 – 71) Reword and expand, something like: “ Sea turtle populations worldwide are in decline because of an assortment of threats, most of which are attributed to anthropogenic pressure including development and loss of nesting habitat, fishing by-catch, poaching, climate change, ocean pollution”.

*This change has been made.*

Line 75) If you make the above change then include “also highly” to this sentence: “Newly hatched sea turtles (hatchlings) are also highly susceptible to…..”

*This change has been made.*

Line 72) “Conservation efforts largely concentrate on protecting juveniles…..” Move this to start of 3rd paragraph and further expand “….because of the survival rates of hatchlings are low (reference)”

*This paragraph has been reworked and the change has been made.*

Lines 339 – 342) Rephase suggested: “Using TurtleSense, motion readings from 79 nests were collected over a period of 5 years….(include numbers as you have done)….From these, the 72 C caretta nests from Hatteras Island were analysed for this study (2014 n=20)…etc.”
*This change has been made.*

Lines 345-346) Rephase suggested: “Graphs were automatically generated with sensor data once a day for the first 40 days and then every four hours after that so that nest data could be analysed for patterns of activity throughout development.”

*This change has been made.*

Line 354) include section about relocated number of nests on line 342 after you give the total nest counts instead of here.

*This section has been moved.*

Line 417) Include % of nests that it did appear in

*This section has been updated to say 1/3 of nests.*

Line 523) I still don’t see where the nest depth description is in the methods. Please include in methods how the measure was taken (to top of clutch).

*This information has been added to the methods (Line 343).*

This sentence should be included in methods not results, and change “is” to “was”:

*This change has been made.*

“Nest depth was recorded when the sensor was placed”

*This change has been made.*

Then under the results section for nest depth, the paragraph can start like: “The mean nest depth was…..”.

*This change has been made (Line 523).*


Line 571) Sub headings here would be useful for each phase, for example:

Incubation: The first period of initial incubation appears to show little motion besides that of background noise.

Prehatch:

Hatching:

Posthatch:

*These subheadings have been added.*

Line 651) “cue” not clue

*This change has been made.*
