# Supplementary material for: The secret life of baby turtles: A novel system to predict hatchling emergence, detect infertile nests, and remotely monitor sea turtle nest events
Source: PLoS One. 2022 Oct 26;17(10):e0275088. doi: 10.1371/journal.pone.0275088 (PMC9605334; doi:10.1371/journal.pone.0275088)
Supplement: Attachment — Submitted filename: reviewer response SEP8_2022.docx [file pone.0275088.s004.docx]

Sept 8, 2022

Dear PLoS ONE,

Thank you to the reviewer for your careful reading of the manuscript. We find your help extremely useful and are grateful for your time. We have made all requested changes, as listed below.

Thank you,

Erin Clabough

Required Changes:

1. Line 69, 120, etc. the authors refer to sea turtles as if they are one species in several places. This needs to be corrected.

*This change has been made, more specifically referring to individual species in current line 69, 118, 119, 124.*

2. Line 72: this is not a complete sentence; please revise.

*This change has been made to include the word “pollution.”*

3. Line 135: informal language used here describing "cork in bottle" situation; please revise.

*This change has been made to remove the informal language in line 139.*

4. Throughout document: "data" is a plural word and needs to be treated that way grammatically at each use in the manuscript. For example, the authors often use "data was" or "data is", when the correct version is "data were" or "data are".

*This change has been made in numerous locations throughout the document.*

5. Throughout the manuscript: the authors often place the punctuation at the end of sentences before the brackets containing the references. This needs to be corrected in every instance.

*This change has been made throughout the document.*
